# Access to mass media messages, and use of family planning in Nigeria: a spatio-demographic analysis from the 2013 DHS

**DOI:** 10.1186/s12889-016-2979-z

**Published:** 2016-05-24

**Authors:** Chukwuedozie K. Ajaero, Clifford Odimegwu, Ijeoma D. Ajaero, Chidiebere A. Nwachukwu

**Affiliations:** Department of Geography, University of Nigeria Nsukka, Nsukka, Nigeria; Demography and Population Studies Programme, University of the Witwatersrand, Johannesburg, South Africa; Department of Mass Communication, University of Nigeria Nsukka, Nsukka, Nigeria

**Keywords:** Family planning, Mass media messages, Nigeria, Spatio-demographic, characteristics

## Abstract

**Background:**

Nigeria has the highest population in sub-Saharan Africa with high birth and growth rates. There is therefore need for family planning to regulate and stabilize this population. This study examined the relationship between access to mass media messages on family planning and use of family planning in Nigeria. It also investigated the impacts of spatio-demographic variables on the relationship between access to mass media messages and use of family planning.

**Methods:**

Data from the 2013 demographic and health survey of Nigeria which was conducted in all the 36 states of Nigeria, and Abuja were used for the study. The sample was weighted to ensure representativeness. Univariate, bivariate and binary logistic regressions were conducted. The relationship between each of the access to mass media messages, and the family planning variables were determined with Pearson correlation analysis.

**Results:**

The correlation results showed significant but weak direct relationships between the access to mass media messages and use of family planning at *p* < 0.0001 with access to television messages (r = 0.239) being associated with highest use of family planning. Some of the results of the adjusted regression analysis showed that access to television messages (OR = 1.2.225; *p* < 0.0001), and radio messages (OR = 1.945; *p* < 0.0001) increase the likelihood of the use of family planning. The adjusted regression model also indicated increased likelihood in the use of family planning by respondents with secondary education (OR = 2.709; *p* < 0.0001), the married (OR = 1.274; *p* < 0.001), and respondents within the highest wealth quintiles (OR = 3.442; *p* < 0.0001).

**Conclusions:**

There exist significant variations within spatio-demographic groups with regards to having access to mass media messages on family planning, and on the use of family planning. The results showed that access to mass media messages increases the likelihood of the use of family planning. Also people with higher socioeconomic status and those from the Southern part of the country make more use of family planning. There is need to improve the socioeconomic status of the populations. Also, the quality and regularity of mass media messages should be improved, while other communication avenues such as traditional institutions, blogs, and seminars for youths should be used to make family planning messages more acceptable.

## Background

Many countries, especially in sub-Saharan Africa face population related problems like spiraling population growth and contracting economy, resulting in situations where nations are unable to muster adequate socio-economic resources to cater to the needs of their citizens [1]. Added to this is the need to promote reproductive health which can be harmed by uncontrolled child bearing, and unprotected sex, leading to avoidable social dislocations. It is in the realization of the importance of keeping tabs on population growth and reproductive health that nations, international agencies and non-governmental organizations (NGOs) spend time and resources to promote family planning.

One of the strategies often employed in the promotion of family planning is the utilization of the mass media to make populations aware of the benefits of the use of family planning. The reason for this is located in the fact that information can have a positive influence on people’s attitude and actions. Research has also shown that depending on the goals of a family planning intervention, such communications can create awareness, increase knowledge and lead to a desired behavioral change [2–12]. For instance, a study in Akwa Ibom, Nigeria found that of the respondents who had been exposed to favorable information about family planning, 46.9 percent had used family planning in contrast with the respondents who were exposed to unfavorable information about family planning, 87.4 percent of who did not use family planning [13]. Also, lack of knowledge and misconceptions about family planning have been strongly linked with non-use of family planning methods [14].

Furthermore, the mass media as credible sources of information have the capacity to raise awareness, increase knowledge level, and influence attitudes towards family planning [1, 15, 16]. Also, another work [6] in Kenya also found that access to media messages affected ever use of contraceptives, current use, intention to use contraceptives and desire for future births. In addition, several studies have focused on how mass media campaigns on family planning have led to improvement in reproductive health in different countries [10, 17–25]. In addition, it has been noted that social marketing could also be used together with the mass media to promote reproductive health [26].

The is also evidence from literature that use of family planning may result to various adverse effects as an increase in paternal age decreases semen quality and prolongs the time of conception for women [27, 28], increases pregnancy complications [29], and results in adverse health outcomes for children [30]. Conversely, family planning has been associated with several benefits. These include reduction in maternal deaths as number of deaths is reduced, and there is a decrease in the number of unsafe abortions from unintended pregnancies [31] even as family planning helps the women to plan their pregnancies and postpone child bearing [32, 33]. Family planning also contributes to gender equality [34] as these women can acquire tertiary education, and increase their chances of employment and higher incomes [35–38].

However, irrespective of the benefits promoted in family planning interventions, people do not respond alike to them. Family planning campaigns may raise awareness about family planning, its benefits and methods without a corresponding rise in the number of people adopting the promoted measures. This is as a result of some intervening variables such as education, marital status, income, and spousal relationships that influence people’s attitude to family planning [39–43]. Furthermore, it has been found that though there was a high level of awareness about family planning in a rural community in Nigeria as a result of a media campaign, the adoption of the new family planning methods remains low [1]. This low uptake of the modern methods was attributed to the desire of the average rural Nigerian household for large family size, and the need to have more male children. This low adoption of modern family planning methods evident in rural Nigeria is also consistent with the findings of other works in Ghana and Nigeria [14, 15]. While educational attainment has a positive relationship with approval of family planning and knowledge of specific methods [14, 15, 44], other predictors of family planning usage in Nigeria include ethnicity, religion, spousal approval and communication with spouse [45].

Consequently, the role of the mass media cannot be ignored in the pursuit of improved maternal and reproductive health in Nigeria. Since studies have shown that the media can influence people positively in adopting family planning methods, it is suggested that “intense campaign exposure, may be necessary to change behavior, but moderate exposure and access to mass media messages may be sufficient to change attitudes and stimulate discussion” [10]. However, a nationally representative empirical study on how mass media messages and spatio-demographic characteristics of the population affect the uptake of family planning in Nigeria is lacking. It is against this backdrop that this study examined the relationship between access to mass media messages on family planning and the use of family planning in Nigeria. It also investigated the impacts of spatio-demographic variables on the relationship between access to mass media messages and family planning, using data from the 2013 demographic and health survey of Nigeria which was conducted in all the 36 states of Nigeria, and Abuja. The findings will be useful in identifying ways of improving the effectiveness of the mass media, and spatio-demographic characteristics in improving the use of family planning in Nigeria.

## Methods

### Data collection

The data for this study is the 2013 Nigeria Demographic and Health Survey (DHS) which is the fourth survey to be implemented by the National Population Commission (NPC) of Nigeria and ICF International USA in association with USAID, UKaid, and UNFPA. The survey was carried out on a nationally representative sample of 38,948 women age 15–49 in 15,859 urban and 22, 663 rural households from all the 36 States and Federal Capital Territory (FCT) of Nigeria. The response rates for this survey among women aged 15–49 years are 97.3 % in the urban areas and 97.8 % in the rural areas. The sampling frame used for the survey was generated from the results of the 2006 Population and Housing Census of Nigeria- the most recent census of Nigeria. The sampling frame for the 2006 census made use of states, in which the states are subdivided into local government areas (LGAs), each LGA subdivided into localities, and each locality subdivided into enumeration areas (EAs). Consequently, the primary sampling unit (PSU) otherwise referred to as a cluster for the DHS was defined on the basis of EAs adopted in the 2006 census. It should also be noted here that these 36 states in Nigeria have been grouped into six geo-political zones based on geographical spread and environmental conditions. The six zones are North Central, North East, North West, South East, South South, and South West. The sample was subsequently selected using a stratified three-stage cluster design made up of 372 urban clusters and 532 rural clusters, totaling 904 clusters in all. In each cluster, 45 households were selected and all the women aged 15–49 who are either permanent residents or visitors present in the households were eligible to be interviewed. Finally, the spatial representation of the data collected from the survey was therefore on the basis geo-political zones, States, and rural/urban residence.

### Variables used for the study

This study utilized three major categories of variables which are further classified into independent and dependent variables. These three variables are; current use of family planning(dependent/outcome variable); access to mass media messages on family planning (independent dummy variables); and spatio-demographic variables (independent variables).

The current use of family planning (dependent/outcome variable) denotes whether traditional or modern methods are used, so long as any type of planning were used by women age 15–49 years. The justification for this is to ascertain the general level of current use of family planning as using only the modern methods of family planning in the analysis may exclude some people who because of their place of residence, wealth index or religious affiliation may not subscribe to certain modern methods of family planning but may be comfortable with the traditional methods despite having access to messages on modern methods of family planning from the mass media. This variable is measured as a dichotomous variable coded as use of family planning method or non-use of family planning method.

In terms of the independent variables, access to mass media messages on the family were examined using messages about family planning got from radio, television or newspaper. These media messages were also measured as dichotomous variables coded as having access or not having access to family planning messages. Finally, the categorical spatio-demographic variables are (i) rural/ urban residence (ii) geopolitical zone/region (iii) five-year age group (iv) education (v) wealth index (vi) employment status, (vii) religion, and (vii) marital status of respondents.

### Data analysis

Before the commencement of data analysis, the dataset was weighted to account for differences due to under-sampling and over sampling as per the survey design using the stata svyset command. Subsequently, the analysis of the data involved univariate analysis of the characteristics of the study population. Also, bivariate analyses of spatio-demographic characteristics and each of the access to mass media messages on family planning variables and the current use of family planning were carried out using Pearson chi-square test. Pearson correlation was used to examine the relationship between each of the access to mass media messages on family planning and the use of family planning services. Finally, binary logistic regressions were used to estimate the access to mass media messages and spatio-demographic predictors of use of family planning in Nigeria. The regression coefficients of the independent variables are expressed as Odds Ratio (OR). A variable with Odds Ratio greater than 1.00 implied that the variable increases the likelihood of the outcome (use of family planning) while it is the opposite when the OR is less than 1.00. Consequently, three regression models are generated for this study namely models 1, 2 and 3. Model 1 is the unadjusted model of current use family planning and each of the independent variables while, in model 2 represents the adjusted model of current use of family planning and all the three variables of access to messages on family planning from radio, television, and newspapers. Finally, Model 3 is the adjusted model of current use of family planning and all the independent variables of access to mass media messages, and the spatio-demographic characteristics.

### Ethical considerations

This study is a secondary analysis of anonymous data from the Nigeria Demographic and Health Survey (DHS) 2013. The survey was approved by the National Health Research Ethics Committee in Nigeria (Approval no: NHREC/01/01/2007). Informed consent was obtained from respondents during the process of data collection. Formal approval to use the data was also obtained from the DHS program.

## Results

### Characteristics of the sample population

A total of 38,948 women aged 15–49 years were interviewed during the Nigeria 2013 Demographic and Health that covered the 36 states and the FCT of Nigeria (Table 1). Generally, the proportion of respondents decreased with increasing age in the number of years while more of the respondents (57.9 %) lived in rural areas as against 42.1 % living in urban areas. These show that the population was relatively a young one even as the majority of the Nigerian populace lived in rural areas. Furthermore, respondents with no education were more in number (14,729) compared to women with secondary education (13,927), and those with more than secondary education (3,558). The respondents across the various wealth index quintiles were relatively equally distributed, but those in the highest wealth index (8,910) constituted the greatest proportion of the population. In terms of spatial distribution, the North West population (30.5 %) had the largest number of respondents while the South East (11.5 %) constituted the smallest proportion of respondents. Finally, both the never married (23.9 %) and the married (69.4 %) together made up more than 90 % of respondents sampled in terms of marital status (Table 1).

**Table 1:** Characteristics of the study population

Spatio-demographic characteristics	Weighted frequency	Weighted percent
Age group		
15–19	7,820	20.1
20–24	6,757	17.3
25–29	7,145	18.3
30–34	5,467	14
35–39	4,718	12.1
40–44	3,620	9.3
45–49	3,422	8.8
Education		
No education	14,729	37.8
Primary	6,734	17.3
Secondary	13,927	35.8
Higher	3,558	9.1
Marital Status		
Never married	9,326	23.9
Married	27,043	69.4
Living with Partner	787	2
Divorced/sep	826	2.1
Widowed	967	2.5
Region		
North Central	5,572	14.3
North East	5,766	14.8
North West	11,877	30.5
South East	4,476	11.5
South South	4,942	12.7
South West	6,314	16.2
Residence		
Urban	16,414	42.1
Rural	22,534	57.9
Wealth Index		
Lowest	7,132	18.3
Second	7,428	19.1
Middle	7,486	19.2
Fourth	7,992	20.5
Highest	8,910	22.9

### Bivariate analyses of access to mass media messages, and the use of family planning

The results of the bivariate and Chi-square analyses revealed that all the socio-demographic characteristics were significantly associated with all the four variables of; current use of family planning; and access to mass media information about family planning from television, from radio, and from newspapers (Table 2). Generally, the results of access to mass media messages show that an increase in socio-economic status of the respondents led to an increase in their probability of getting access to family planning messages from the various mass media. For instance, while 13.45 % of people with no education had access to radio messages, 67.55 % of respondents with post-secondary education got information about family planning from the radio. In terms of the regions of respondents, 14.62 % of people from North East got information from the radio as against 63.66 % from the South West. Also, 8.29 % of people in the lowest wealth quintile had access to radio information compared to 57.84 % from the highest wealth quintile.

**Table 2:** Bivariate and Chi-Square analyses of access to mass media messages, and use of family planning

Spatio-demographic	Radio			TV			N P			FP		
characteristics	yes	%	sig.	yes	%	sig.	yes	%	sig.	yes	%	sig.
Age group												
15–19	1,911	24.20	0.000	1,088	13.79	0.000	352	4.47	0.000	539	6.82	0.000
20–24	2,167	32.31		1,324	19.74		508	7.59		1,192	17.75	
25–29	2,538	36.09		1,635	23.26		585	8.83		1,260	17.91	
30–34	1,992	37.12		1,280	23.86		466	8.70		1,033	19.23	
35–39	1,734	36.89		1,060	22.58		365	7.78		976	20.76	
40–44	1,270	34.69		812	22.19		286	7.82		776	21.18	
45–49	1,146	32.25		671	18.90		205	5.77		449	12.63	
Education												
No education	1,846	13.45	0.000	331	2.41	0.000	32	0.23	0.000	400	2.91	0.000
Primary	2,214	31.17		1,120	15.78		194	2.74		1,210	17.03	
Secondary	6,204	43.10		4,246	29.51		1,334	9.29		3,257	22.61	
Higher	2,494	67.55		2,173	58.87		1.207	32.75		1,358	36.73	
Marital Status												
Never married	8,209	31.11	0.000	4,714	17.88	0.000	1,507	5.72	0.000	3,854	14.6	0.000
Married	395	45.45		292	33.56		77	8.87		249	28.59	
Living with Partner	347	34.94		202	20.36		68	6.87		96	9.67	
Divorced/sep	162	37.76		106	24.71		42	9.84		97	22.61	
Widowed	120	27.78		49	11.34		21	4.86		35	8.10	
Religion												
Traditionalist	31	8.81	0.000	20	5.68	0.000	5	1.42	0.000	24	6.82	0.000
Christianity	8,169	41.21		6,047	30.52		2,276	11.50		5,004	25.22	
Islam	4,505	24.27		1,776	9.57		473	2.55		1,174	6.32	
Employment status												
Not working	12,379	33.34	0.000	7,649	20.61	0.000	2.671	7.21	0.000	6,039	16.26	0.000
Working	209	19.24		135	12.44		47	4.33		86	7.92	
Region												
North Central	1,871	29.96	0.000	1,205	19.30	0.000	512	8.21	0.000	1,019	16.30	0.000
North East	968	14.62		469	7.09		165	2.50		256	3.86	
North West	2,082	21.54		370	3.83		151	1.56		334	3.45	
South East	1,725	38.69		1,042	23.38		511	11.47		1,168	26.18	
South South	2,375	39.22		1,886	31.19		737	12.22		1,590	26.25	
South West	3,737	63.66		2,898	49.37		691	11.78		1,858	31.63	
Residence												
Urban	7,600	48.93	0.000	5,526	35.59	0.000	2.037	13.14	0.000	3,704	23.83	0.000
Rural	5,158	22.06		2,344	10.03		730	3.13		2,521	10.77	
Wealth Index												
Lowest	547	8.29	0.000	41	0.62	0.000	14	0.21	0.000	121	1.83	0.000
Second	1,176	15.65		212	2.83		63	0.84		456	6.07	
Middle	2,284	28.58		984	12.32		307	3.85		1,166	14.57	
Fourth	3,910	46.29		2,517	29.81		705	8.36		1,883	22.28	
Highest	4,841	57.84		4,116	49.19		1.678	20.10		2,599	31.01	

*FP* Use of family planning

*TV* Television messages on family planning

*Radio* Radio messages on family planning

*NP* Newspaper messages on family planning

*Sig* Chi Square significance level

Access to family planning information from television varies significantly among the various socio-demographic groups as 10.03 % of rural residents could access family planning information on television as against 35.59 % or urban residents. In terms of marital status, 11.34 % of the widowed had access compared to 33.56 % of married respondents. Other socio-demographic groups with relatively higher access to television messages about family planning include people with higher education (58.87 %), people from South West (49.37 %), and people within the highest wealth quintile (49.19 %). In terms of access to newspaper information on family planning the following had relatively lower access; the 15–19 years age group (4.47 %), no education group (0.23 %), the widowed (4.86 %), the working population (4.33 %), North West residents (1.56 %), rural populations (3.13 %) and populations within the lowest wealth quintile (0.21 %).

The use of family planning services also varied across the socio-demographic groups as indicated in Table 2. The use of family planning services increased with an increment in the age of respondents as only 6.82 % of the 15–19 age-group as against 21.18 % of the 40–44 age-group use family planning services. The proportion of users also got reduced to 12.63 % for the 45–49 age-group. The other groups that made relatively greater use of family planning services in the country are; people with higher education (36.73 %), the married (28.59 %), people from South West (31.63 %), urban residents (23.83 %), and people with the highest wealth quintile (31.01 %).

### Do access to mass media messages on family planning correlate with use of family planning?

The Pearson correlation analysis of mass media messages on family planning and utilization of family planning methods generally indicated weak but very significant direct relationships. This means that as access to messages about family planning from the various mass media increases, the use of family planning also increases in the country. Specifically, the greatest correlation for the mass media and use of family planning was found for television messages (r = 0.239; *p* < 0.0001) followed by radio messages (*r* = 0.216; *p* <0.0001) while the least correlation was found for access to newspaper messages (r = 0.156; *p* < 0.0001). These correlation coefficients show that generally, access to family planning messages using these three mass media has been effective in influencing the use of family planning. However, the comprehension of the magnitude of the impacts of these mass media messages, and the spatio-demographics characteristics on use of family planning is necessary in order to have an empirical basis for further designing and implementation of strategies that could enhance their effectiveness in influencing the use of family planning methods in Nigeria. Consequently, the regression analyses were used to highlight the magnitude of the impacts of mass media messages and other spatio-demographic characteristics on the use of family planning methods in Nigeria.

### The predictors of use of family planning in Nigeria

The regression results for the predictors of the use of family planning are shown in Table 3. In model 1 which depicts the unadjusted regression coefficients, it can be seen that the odds ratio of most of the independent variables increased the likelihood of respondents making use of family planning methods. The only variables that their odds ratio decreased the likelihood of respondents using family planning are the respondents living with a partner (OR = 0.626; *p* < 0.0001), the widowed (OR = 0.516; *p* < 0.0001), the Islam religion respondents (OR = 0.922), North East residents (OR = 0.206;*p* < 0.0001), North West residents (OR = 0.184;*p* < 0.0001), respondents who were not working (OR = 0.443; *p* < 0.0001), and the rural dwellers (OR = 0.386; *p* < 0.0001). Within each of the categories of independent variables (mass media, age group, marital status etc), the following relatively predicted more use of family planning. They are access to television messages (OR = 3.380; *p* < 0.0001), 40–44 age-group (OR = 3.673; *p* < 0.0001), respondents with post-secondary education (OR = 19. 363; *p* < 0.0001), the married (OR = 2.342; *p* < 0.0001), the Christian respondents (OR = 4.610; *p* < 0.000), the South West residents (OR = 2.375; *p* < 0.0001), and the respondents in the highest wealth index (OR = 24.080; *p* < 0.0001).

**Table 3:** Regression results of the predictors of current use of family planning

Regression variables	Model 1	Model 2	Model 3
Intercept	0.190***	0.112***	0.012***
Access to radio messages	3.225***	1.945***	1.359***
Access to television messages	3.830***	2.221***	1.031
Access to newspaper messages	3.434***	1.360***	1.120
Age group			
15–19	RC		RC
20–24	2.950***		2.120***
25–29	2.981***		2.460***
30–34	3.253***		3.275***
35–39	3.581***		4.135***
40–44	3.673***		4.454***
45–49	1.976***		2.583***
Education			
No education	RC		RC
Primary	6.847***		2.344***
Secondary	9.741***		2.709***
Higher	19.363***		2.474***
Marital Status			
Never married	RC		RC
Married	2.342***		1.274**
Living with Partner	0.626***		0.359***
Divorced/sep	1.709***		0.958
Widowed	0.516***		0.604*
Religion			
Traditionalist	RC		RC
Christianity	4.610***		1.579*
Islam	0.922		0.985
Employment status			
Not working	RC		RC
Working	0.443***		0.802
Region			
North Central	RC		RC
North East	0.206***		0.387***
North West	0.184***		0.395***
South East	1.821***		0.986
South South	1.827***		0.812**
South West	2.375***		1.294***
Residence			
Urban	RC		RC
Rural	0.386***		0.852**
Wealth Index			
Lowest	RC		RC
Second	3.460***		1.530***
Middle	9.137***		2.276***
Fourth	15.358***		2.797***
Highest	24.080***		3.442***

*RC* Reference category

* *p* < 0.05; ** *p* < 0.001; **** *p* < 0.000

In model 2 in which use of family planning was adjusted for access to mass media messages, the results still showed that access to mass media messages significantly increased the likelihood of respondents making use of family planning with access to television message (OR = 2.221;*p* < 0.0001) still predicting 2.221 times more of the likelihood of use family planning compared to radio message which was 1.945 times likely to increase use of family planning and newspaper message which was 1.360 times more likely to increase use of family planning. The results also showed that the combined effects of the various mass media messages in the adjusted model reduced their odds ratios relative to their odds ratios in the unadjusted model but still increased the likelihood of respondents making use of family planning. For instance, while the access to radio messages was 3.225 more likely to lead to use of family planning in model 1, it was 1.945 more likely to lead to use of family planning in model 2.

Model 3 shows the combined effects of the independent variables of access to mass media messages and the spatio-demographic characteristics on the dependent variable. The results show that all the variables within each category of the independent variables (mass media, age group, marital status etc) which predicted more likelihood of use of family in the unadjusted model also predicted more likelihood of use of family planning in the adjusted model. The exception to this pattern exists in the mass media category where television messages predicted more likelihood in both the unadjusted model and in the adjusted Model 2 but access to radio massages (OR = 1.359; *p* < 0.0001) predicted more likelihood in adjusted model 2. Also in the education category, post-secondary education (OR = 19.363;*p* < 0.0001) predicted more likelihood in the unadjusted model but in the adjusted model, secondary education respondents (OR = 2.709;*p* < 0.0001) predicted more likelihood of use of family planning. Generally however, model 3 shows that most of the odds ratios of independent variables had lower values compared to the unadjusted values. The variables with increased odds ratios in the adjusted model relative to the unadjusted model are the 30–34 age group (OR = 3.275; *p* < 0.0001), the widowed (OR = 0.604; *p* < 0.0001), Islam religion respondents (OR = 0.985), the employed respondents (OR = 0.802), and the rural dwellers (OR = 0.852; *p* < 0.001).

## Discussion

This study found that access to mass media messages increased the likelihood of respondents making use of family planning. This means that access to information on family planning is effective in positively influencing peoples’ attitude towards use of family planning in Nigeria and this results supports earlier findings by other researchers who posited that mass media messages on family planning was effective in increasing the use of family planning [1, 16, 25]. The results of both the bivariate and regression analyses also showed that increase in socioeconomic status leads to a corresponding increase in the use of family planning. These results agree with other findings which showed that people with higher educational qualification are more like to make use of family planning as they are better informed of its importance, and also because they need to use family planning to ensure that their educational pursuits are not truncated by child bearing [36, 41]. Also as the wealth index increases from the lowest category, so also the more the likelihood of their odds ratios predicting use of family planning. In other words, as the wealth of households increase, the household members are more likely to make use of family planning so as to ensure a better quality of life for the household members. This finding is also corroborated by earlier studies which posited that higher socioeconomic status of households leads to higher uptake of family planning [14, 40, 44].

Television messages have the greatest impact on the tendency for people to use family planning, and it is followed in importance by radio messages. The reduction in the explanatory power of the mass media in influencing use of family planning, when spatio-demographic characteristics are added in model 3, shows that the spatio-demographic variables also influence use of family planning by affecting how these mass media messages are received and utilized by respondents. This finding is in agreement with other studies in which they note that other socio-demographic variables such as place of residence, marital status, and employment status play a vital role in determining the use or otherwise of family planning even in the presence of family planning awareness among populations [17–19]. For instance, our findings show that rural respondents are less likely to make use of family planning compared to urban respondents. This also may due to the fact that the urban dwellers have better access to family planning messages (Table 2), and as such has more knowledge about family planning [1].

The results of the regression analyses also revealed significant and positive odds ratios between use of family planning and respondents of all age groups relative to the reference category of the 15–19 age group. This means that increase in age groups significantly increase the likelihood of the use of family planning with the exception of the 45–49 age group. Despite the fact that the odds ratios of this age-group for model 1 (OR = 1.976; *p* < 0.0001), and for model 3 (OR = 2.583; *p* < 0.0001) increases the likelihood of use of family planning, odds ratios were less than the preceding age group. This indicates that even though, this age group still makes use of family planning, their propensity to make use of family planning decreases. This reduction in use of family planning by the 45–49 age-group is due to the fact that most of them have reached menopause, stopped child bearing, and subsequently, the need for use of family planning becomes minimal [42].

The married and the divorced/separated are more likely to make use of family planning relative to the reference category of the never married. Literature show that married respondents are always sexually active and thus are more likely to use family planning to regulate their rate of becoming pregnant especially when their babies are still young [14, 15, 39–43]. With regards to the geo-political zones of the country, the regions that are less likely to make use of family planning are North East, South East and South South while the South West respondents are more likely to make use of family planning, with North Central as the reference category. The reason may be because the Northern part of Nigeria are highly populated have more expanse of land for population expansion unlike the Southern part that has high population densities. Secondly, the religion practices in the Northern parts of Nigeria which encourage polygamy and does noes encourage family planning. This is at variance with what happens in Southern parts of Nigeria where Christianity encourages people to marry one wife. Consequently, the results show that culture (including religion) and place of residence influence use of family planning [1, 14]. In addition, the results in Table 2 shows that the Northern parts of Nigeria has less access to mass media messages on family planning compared to their Southern counterparts, and as such are less enlightened about the importance of family planning.

## Conclusions

The study showed significant variations within spatio-demographic groups with regards to having access to mass media messages on family planning, and on how they make use of family planning. For instance, Northern Nigeria populations had less access to mass media messages on family planning messages and were also less likely to use family planning, compared to populations in Southern Nigeria. Also, people with lower socio-economic status had less access to mass media and were less likely to use family planning. The correlation results indicated that mass media messages significantly increased use of family planning. Both the bivariate and regression results showed that access to television messages influenced use of family planning more than messages from the other mass media. The regression results also showed that in addition to the influence of mass media in encouraging use of family planning, other spatio-demographic variables such as higher educational qualification, increase in age of women, being married, being a from the Southern part of Nigeria, and increase in the wealth of households are the most significant characteristics that encouraged use of family planning in Nigeria. Based on the findings, wider coverage area and improved quality of mass media messages on family planning should be implemented especially in the Northern part of Nigeria. Also, there is the need for improvement in educational attainment of women as it will help to enlighten them on the importance of family planning while at the same time delaying their age of first birth. It will also be necessary to put in place infrastructural facilities such as electricity, and skills acquisition centers that will also create employments especially for the youths, who represent the reproductive cohorts of the population. While employment and skills acquisition will help to ensure that the populace is appropriately engaged in productive economic activities, access to electricity will also help them have access to information about the utility of family planning through various mass media sources. Finally, other communication avenues such as traditional institutions, blogs, and seminars for youths as means of making family planning more acceptable to the population should be explored.

### Data availability

The dataset(s) supporting the conclusions of this article is(are) available in the Demographic and Health Survey repository, in http://dhsprogram.com/data/dataset/Nigeria_StandardDHS_2013.cfm?flag=1.

## Abbreviations

DHS: Demographic and Health Survey
EA: enumeration area
FCT: Federal Capital Territory of Nigeria (Abuja)
ICF: Inner City Fund International
LGA: Local Government Area
NPC: National Population commission of Nigeria
NGO: Non-governmental Organization
OR: odds ratio
PSU: primary sampling unit
UKaid: United Kingdom Agency for International Development
USA: United States of America
USAID: United States Agency for International Development
